# Measurement of ATGL activity using adiposomes

**DOI:** 10.52601/bpr.2023.220016

**Published:** 2023-02-28

**Authors:** Xuejing Ma, Zelun Zhi, Shuyan Zhang, Pingsheng Liu

**Affiliations:** 1 National Laboratory of Biomacromolecules, CAS Center for Excellence in Biomacromolecules, Institute of Biophysics, Chinese Academy of Sciences, Beijing 100101, China; 2 University of Chinese Academy of Sciences, Beijing 100049, China; 3 Department of Life Sciences, Cangzhou Normal University, Cangzhou 061001, Hebei, China; 4 Institute of Infectious Diseases, Beijing Key Laboratory of Emerging Infectious Diseases, Beijing Ditan Hospital, Capital Medical University, Beijing 100015, China; 5 Beijing Institute of Infectious Diseases, Beijing 100015, China

**Keywords:** ATGL, Enzymatic activity, Adiposome, Lipid droplet

## Abstract

Adipose triacylglycerol lipase (ATGL) is a dynamic lipid droplet-associated protein involved in cellular lipolysis, which is conserved from bacteria to humans. Recent methods that measure the enzymatic activity of ATGL *in vitro* are established using lipid emulsions. However, the lipid emulsion platforms contain various membranous structures which reduce the accuracy of enzymatic activity determination. Therefore, a new platform and corresponding method are required for accurate measurement of ATGL enzymatic activity that represents cellular lipid and energy homeostasis. Adiposomes are artificial lipid nanostructures mimicking lipid droplets. Employing adiposome as a platform, we have developed an assay to measure the enzymatic activity of ATGL* in vitro*. Here, a detailed protocol is described to explain how to measure the activity of ATGL using adiposomes. This method successfully proves the concept of lipid droplet-mimetic lipase activity determining platform and provides a tool to identify the active sites of lipases.

## INTRODUCTION

Ectopic storage of lipids in non-adipose tissues can lead to human metabolic syndromes, *e.g.*, obesity, type 2 diabetes, fatty liver diseases, and atherosclerosis (Samuel and Shulman 2012). Therefore, it is essential to understand the intracellular lipid accumulation and the regulation of lipid metabolism. Lipid droplet (LD) is an organelle storing neutral lipids which is composed of a neutral lipid core, surrounded by a phospholipid monolayer membrane and various peripheral proteins (Farese and Walther 2009). It is also a hub to regulate the intracellular lipid metabolism including lipid synthesis, lipolysis, and lipid transport (Olzmann and Carvalho 2019). Triacylglycerols (TAGs) are one class of the main components of LDs and therefore TAG metabolism-related enzymes can be enriched and active on the surface of LDs (Wilfling* et al.* 2013).

ATGL, also known as patatin-like phospholipase domain-containing protein 2, is one significant lipase which catalyzes the hydrolysis of TAG to fatty acids and diacylglycerols (Zimmermann* et al.* 2004). It has been found to localize on LDs and plays a key role in LD degradation (Smirnova* et al.* 2006). The reduction of ATGL on LDs can cause excessive accumulation of lipids in various tissues and further lead to metabolic disorders. For example, ATGL knockout results in excessive accumulation of lipids in the heart of a mouse (Haemmerle* et al.* 2006). Moreover, ATGL is down-regulated on LDs from the cardiomyocytes of heart failure rats (Li* et al.* 2016). In A549 cells, deletion of ATGL leads to TAG accumulation in LDs and high levels of cellular phospholipids and bioactive lipid species (Tomin* et al.* 2018). In nephrocyte, ATGL overexpression can rescue the high-fat diet-induced accumulation of LDs as well as the defects in renal endocytosis (Lubojemska* et al.* 2021). In hepatocytes, ATGL deficiency causes steatosis, while the low hepatic lipolysis and increased PPARδ activity may counteract hepatic inflammation and ER stress (Fuchs* et al.* 2021). Therefore, understanding the role of ATGL is essential for the therapeutic studies of human metabolic diseases.

Phosphorylation is one of the significant modifications regulating the activity and subcellular location of ATGL (Grabner* et al.* 2021; Xie* et al.* 2014; Zimmermann* et al.* 2004). Two phosphorylation sites of human ATGL, S404 and S428, are first discovered by mass spectrometry techniques (Bartz* et al.* 2007b). For many organisms, the activity of ATGL is also affected by phosphorylation modification. Nematode ATGL-1 is significantly inactivated by AMP-activated protein kinase (AMPK)-mediated phosphorylation at S303 and the life span of *C. elegans* is extended (Narbonne and Roy 2009). In HEK293 cells, phosphorylation of human ATGL by AMPK at S406 increases the TAG hydrolase activity (Ahmadian* et al.* 2011), while murine adipose tissue-specific AMPK knockout causes defective phosphorylation of ATGL at S406 to decrease its TAG hydrolase activity (Kim* et al.* 2016). Protein kinase A (PKA)-mediated phosphorylation of murine ATGL at S396 only increases lipolysis *in vitro* (Pagnon* et al.* 2012; Zimmermann* et al.* 2004). Among the eight phosphorylation sites of ATGL identified in murine adipocytes (Bartz* et al.* 2007a; Schreiber* et al.* 2019; Xie* et al.* 2014), T372 phosphorylation leads to a decreased LD association of ATGL under both basal and stimulated conditions (Xie* et al.* 2014). In HL-1 cells, excessive accumulation of TAG might be related to decreased phosphorylation levels of S406 (Li* et al.* 2021). Hitherto, nine phosphorylation sites of ATGL have been identified and S406 has been verified to regulate its activity (Ahmadian* et al.* 2011; Bartz* et al.* 2007b; Pagnon* et al.* 2012; Xie* et al.* 2014). However, the role of other phosphorylation sites in activity regulation remains unclear. Meanwhile, active ATGL can reduce the volume of LDs and the intracellular protein–protein interactions cannot be excluded to analyze the activity of ATGL, which makes the characterization of ATGL activity challenging.

The enzymatic activity of ATGL is typically measured using emulsions, micelles, or purified LDs (Duncan* et al.* 2008; Rajan* et al.* 2021; Schweiger* et al.* 2008; Zimmermann* et al.* 2004). Compared to the phospholipid monolayer of LDs, the lipid interfaces of the synthetic substrates are heterogeneous (Wang* et al.* 2016). The irregular structures in lipid emulsions or micelles might affect ATGL activity measurement. Besides, for the purified LDs, the lipids in natural LDs are difficult to be manipulated, making it challenging to study the effect of specific lipids on regulating ATGL activity. Moreover, there are multiple proteins on the surface of purified LDs, therefore protein interactions cannot be excluded. The structure of adiposomes, in contrast, is close to that of LDs, and the lipid composition of adiposomes can be precisely changed.

Adiposomes are able to mimic the structure of LDs and further mimic the functions of LDs when recruiting corresponded proteins on adiposomes (Wang* et al.* 2016). Therefore, adiposomes have been applied to perform the *in vitro* assays for LD studies (Lange* et al.* 2021; Ma* et al.* 2021; Zhang* et al.* 2017). Compared with methods to prepare the LD-like emulsions previously reported, the new method separates the adiposome from the impurities so that the resulting adiposome effectively mimics the actual LDs (Chen* et al.* 2015; Fei* et al.* 2011; Krahmer* et al.* 2011; Thiam* et al.* 2013; Tzen and Huang 1992; Zhi* et al.* 2022). Therefore, the adiposome platform is an ideal *in vitro* system to study ATGL activity and even other LD-associated lipases. Here, we present a detailed protocol to describe the measurement of ATGL activity using the adiposome platform.

## MATERIALS AND EQUIPMENT

The reagents, plasmids, buffers, equipment, and software used in this study are listed in Table 1.

**Table 1:** Materials and equipment used in this protocol

Materials or equipment	Source	Identifier
Reagent		
dNTP Mix	GenStar	Cat#A113-01
DOPC^*a*^	Avanti Polar Lipids	Cat#850375
DOPE^*b*^	Avanti Polar Lipids	Cat#850725
DpnI	New England Biolabs	Cat#R0176
EasyPfu DNA Polymerase	TransGen Biotech	Cat#AP111-01
IPTG^*c*^	Amresco	Cat# N679-10G
Liver PtdIns^*d*^	Avanti Polar Lipids	Cat#840042
OptiPhase Supermix Scintillation Cocktail	PerkinElmer	Cat#1200-439
Phusion High-Fidelity DNA Polymerase	New England Biolabs	Cat#M0530L
Pierce^TM^ BCA Protein Assay Kit	Thermo Fisher Scientific	Cat#23225
PMSF^*e*^	Sigma-Aldrich	Cat#52332
T4 DNA Ligase	Takara	Cat#2011B
TAG^*f*^	Extracted from rat fat pad in the laboratory	N/A
TOP10 Competent Cell	TianGen	Cat# CB104
Transetta (DE3) Competent Cell	TransGen Biotech	Cat#CD801-02
Triolein [9, 10-^3^H(N)]	PerkinElmer	Cat#NET431001MC
Tryptone	OXOID	Cat# LP0042B
Yeast Extract Power	OXOID	Cat#LP0021T
Buffers or solutions		
2× Sample Buffer	100 mmol/L Tirs-HCl, 8% SDS (*m*/*v*), 20% glycerol (*v*/*v*), 0.2% Bromophenol blue (*m*/*v*), 200 mmol/L DTT^*g*^ (pH 6.8)	N/A
2× YT^*h*^ Medium	16 mg/mL Tryptone, 10 mg/ mL Yeast extract, 5 mg/mL NaCl	N/A
Buffer B	20 mmol/L HEPES^*i*^, 100 mmol/L KCl, 2 mmol/L MgCl_2_ (pH 7.4)	N/A
Coomassie Brilliant Blue Dye Solution	1 mg/mL Coomassie Brilliant Blue R-250, 45% CH_3_OH, 45% ddH_2_O, 10% CH_3_COOH	N/A
Extraction Solution I	Methanol/chloroform/n-hexane=10:9:7 (*v*/*v*/*v*), stored light-protected at 4°C, prewarm solution before use	N/A
Extraction Solution II	0.1 mol/L K_2_CO_3_ (pH 10.5, adjusted with saturated H_3_BO_3_)	N/A
Solution A	0.25 mol/L Sucrose, 1 mmol/L EDTA, 1 mmol/L DTT, 0.5 mmol/L PMSF	N/A
Tris-NaCl Buffer	50 mmol/L Tris-HCl, 150 mmol/L NaCl (pH 7.4)	N/A
Plasmid		
pET-28a-SMT3-N	Gift from Dr. Sarah Perret	N/A
Equipment		
Centrifuge (5417R, 5424R)	Eppendorf	Cat#10148204
Gamma Meter	PerkinElmer	Cat#2470-0010
Vortex Oscillator	Scientific Industries	N/A
Water Bath	Shanghai Yiheng Scientific Instrument	N/A
Software		
Adobe Illustrator CS5	Adobe	https://www.adobe.com
GraphPad Prism 7.0	GraphPad Software	https://www.graphpad.com/
ImageJ	National Institutes of Health	https://imagej.nih.gov/ij/
Vector NTI	Thermo Fisher Scientific	https://www.thermofisher.cn/cn/zh/home/brands/invitrogen.html
^*a*^ DOPC, 1,2-dioleoyl-sn-glycero-3-phosphocholine; ^*b*^ DOPE, 1,2-dioleoyl-sn-glycero-3-phosphoethanolamine; ^*c*^ IPTG, Isopropyl β-D-1-thiogalatopyranoside; ^*d*^ PtdIns, L-α-phosphatidylinositol; ^*e*^ PMSF, Phenylmethylsulfonyl fluoride; ^*f*^ TAG, Triacylglycerol; ^*g*^ DTT, Dithiothreitol; ^*h*^ YT, Yeast extract-Tryptone; ^*i*^ HEPES, 4- (2-hydroxyerhyl) piperazine-1-erhanesulfonic acid		

## OVERVIEW OF THE EXPERIMENTAL DESIGN

This protocol includes four sections. The first section provides a detailed procedure to construct ATGL expression vectors. The second section presents the steps for the expression of recombinant ATGL. The third section introduces the method to prepare adiposomes. The last section describes the method to measure ATGL activity using adiposomes.

First, ATGL fusion protein expression vector and ATGL mutants are constructed. At present, nine phosphorylation sites of ATGL have been discovered. Although S47 is not a phosphorylation site, it plays a key role in the enzymatic activity (Duncan* et al.* 2010). Therefore, 10 sites are mutated to A and D respectively to investigate the regulation of ATGL activity by each site. Second, ATGL fusion protein as well as ATGL mutant proteins are expressed by *E. coli* expression system. Since ATGL fusion proteins tend to aggregate and precipitate, an SMT3 tag is inserted behind the 6× His sequence but ahead of the N-terminal region of ATGL to improve the solubility. Moreover, the enzymatic activity of ATGL is found to be highly sensitive to imidazole, which means that the protein purification based on affinity chromatography would result in the loss of activity. Therefore, bacterial lysates enriched in ATGLs instead of purified ATGL proteins are extracted for subsequent enzymatic assay. Third, adiposomes are prepared with ^3^H-labeled triolein. Radiolabeled triolein is mixed with normal TAG as the source of the neutral lipid core of adiposomes. Fourth, ATGL fusion protein and mutant proteins are recruited to radiolabeled adiposomes. The free fatty acids (FFAs) generated by TAG hydrolysis are extracted and analyzed for determining radioactivity and calculating the enzymatic activity of ATGLs.

## STEP-BY-STEP PROCEDURE

### Plasmid construction [TIMING 1 week]

1 Construction of SMT3-ATGL fusion protein expression vector.

(A) Design the primers using Vector NTI. Cloning primers are listed in Table 2.

**Table 2:** The primers used in this protocol

Primer	Sequence
SMT3-ATGL -F	GGCGAAGCTTGCATGTTCCCGAGGGAGACCAA
SMT3-ATGL -R	TTATAGCGGCCGCTCAGCAAGGCGGGAGGCCAG
S47A -F	CACATCTACGGAGCCGCGGCAGGGGCG
S47A -R	CGCGGCTCCGTAGATGTGAGTGGCGTTG
S47D -F	CACATCTACGGAGCCGACGCAGGGGCGC
S47D -R	GTCGGCTCCGTAGATGTGAGTGGCGTTG
S87A -F	CCTCTGCATCCCGCGTTCAACCTGGT
S87A -R	CGCGGGATGCAGAGGACCCAGGAACC
S87D -F	CCTCTGCATCCCGACTTCAACCTGGT
S87D -R	GTCGGGATGCAGAGGACCCAGGAACC
T101A -F	TGTCTACTAAAGGCGCTGCCTGCTGA
T101A -R	CGCCTTTAGTAGACAGCCACGGATG
T101D -F	TGTCTACTAAAGGACCTGCCTGCTGA
T101D -F	GTCCTTTAGTAGACAGCCACGGATG
T210A -F	CGCGTCACCAACGCGAGCATCCAGTT
T210A -R	CGCGTTGGTGACGCGAAGCTCGTGGA
T210D -F	CGCGTCACCAACGACAGCATCCAGTT
T210D -R	GTCGTTGGTGACGCGAAGCTCGTGGA
T372A -F	ATGAAAGAGCAGGCGGGTAGCATCT
T372A -R	CGCCTGCTCTTTCATCCACCGGATA
T372D -F	ATGAAAGAGCAGGACGGTAGCATCT
T372D -R	GTCCTGCTCTTTCATCCACCGGATA
S393A -F	GACCATCTGCCTGCGAGACTGTCTGA
S393A -R	CGCAGGCAGATGGTCACCCAATTTC
S393D -F	GACCATCTGCCTGACAGACTGTCTGA
S393D -R	GTCAGGCAGATGGTCACCCAATTTC
Y378A -F	AGCATCTGCCAGGCGCTGGTGATGA
Y378A -R	CGCCTGGCAGATGCTACCCGTCTGCT
Y378D -F	AGCATCTGCCAGGACCTGGTGATGA
Y378D -R	GTCCTGGCAGATGCTACCCGTCTGCT
S396A -F	CCTTCCAGACTGGCGGAGCAGGTGGA
S396A -R	CGCCAGTCTGGAAGGCAGATGGTCA
S396D -F	CCTTCCAGACTGGACGAGCAGGTGGA
S396D -R	GTCCAGTCTGGAAGGCAGATGGTCA
S406A -F	CTGCGACGTGCCCAGGCGCTGCCCTCTG
S406A -R	CGCCTGGGCACGTCGCAGTTCCACCTGC
S406D -F	CTGCGACGTGCCCAGGACCTGCCCTCTG
S406D -R	GTCCTGGGCACGTCGCAGTTCCACCTGC
S430A -F	GTACGAAACAACCTCGCGCTGGGGGACG
S430A -R	CGCGAGGTTGTTTCGTACCCAGTTGGGT
S430D -F	GTACGAAACAACCTCGACCTGGGGGACG
S430D -R	GTCGAGGTTGTTTCGTACCCAGTTGGGT

(B) Amplify ATGL by PCR using cDNA of C2C12 cells as the template. The reaction components and thermocycling conditions of ATGL amplification are listed in Table 3 and Table 4.

**Table 3:** Reagents of PCR reaction

Component	Volume
5× EasyPfu buffer	10 µL
2.5 mmol/L dNTPs	6 µL
10 µmol/L forward primer	1 µL
10 µmol/L reverse primer	1 µL
Template DNA	1.5 µL
Nuclease-free water	30 µL
EasyPfu polymerase	0.5 µL
Total	50 µL

**Table 4:** Procedures for amplification PCR

Step	Temperature	Time
Initial denaturation	94 °C	5 min
25–35 Cycles	94 °C 45–72 °C 72 °C	40 s 40 s 60 s per kb
Final extension	72 °C	10 min
Hold	4 °C	Forever

(C) Collect the PCR product by agarose gel electrophoresis. Digest the PCR product and pET-28a-SMT3-N plasmid with restriction enzymes in 30 °C or 37 °C water bath for 4 h. The digestion mix is listed in Table 5.

**Table 5:** Reagents of digestion reaction

Component	Volume
10× buffer	5 µL
PCR product/plasmid	40 µL/1 µL diluted in 39 µL Nuclease-free water
Restriction enzyme 1	2.5 µL
Restriction enzyme 2	2.5 µL
Total	50 µL

(D) Collect the digestion product by agarose gel electrophoresis and conduct a ligation reaction for 12 h in 16 °C water bath. The ligation mix is listed in Table 6.

**Table 6:** Reagents of ligation reaction

Component	Volume
10× T4 ligation buffer	2 µL
PCR product	15 µL
Plasmid	2 µL
T4 ligase	1 µL
Total	20 µL

(E) Transform the ligation mix into TOP10 competent cells.

(F) Pick three clones and culture them separately in 500 µL resistant LB medium at 37 °C for 2–3 h with 200 r/min shaking for sequencing.

2 Mutagenesis of SMT3-ATGL fusion protein expression vector

(A) Design the primers using Vector NTI. Mutagenesis primers are listed in Table 2.

(B) Conduct single site-directed mutation of ATGL. The reaction components and thermocycling conditions are listed in Table 7 and Table 8.

**Table 7:** Reagents of site-directed mutation

Component	Volume
5× Phusion HF buffer	10 µL
10 mmol/L dNTPs	1 µL
10 µmol/L forward primer	2.5 µL
10 µmol/L reverse primer	2.5 µL
DMSO	1.5 µL
Template DNA	1.5 µL
Nuclease-free water	30.5 µL
Phusion DNA polymerase	0.5 µL
Total	50 µL

**Table 8:** Procedures for site-directed mutation

Step	Temperature	Time
Initial denaturation	98 °C	30 s
25–35 cycles	98 °C 45–72 °C 72 °C	5–10 s 10–30 s 15–30 s per kb
Final extension	72 °C	5–10 min
Hold	4 °C	Forever

(C) Add 1 μL Dpn1 in the PCR products and incubated in 37 °C water bath for 1 h.

(D) Transform 1 μL of the digested products into TOP10 competent cells.

(E) Pick three clones and culture them separately as described in Step 1-F for sequencing.

We find that ATGL is cloned into the SMT3-pET28a expression vector with a 6× His tag and an SMT3 tag ahead of the N-terminal. Through site-directed mutation of ATGL, we construct the mutants of reported phosphorylation sites. Mutation to amino acid residue A mimics the unphosphorylated type, and mutation to D mimics the phosphorylated type.

**[CRITICAL STEP]** (1) Dilute cDNA for PCR reaction. (2) Add enzyme in the last step. (3) Mix and centrifuge the mixture before reaction.

### Protein expression [TIMING 2**–**3 days]

**Figure 1:**
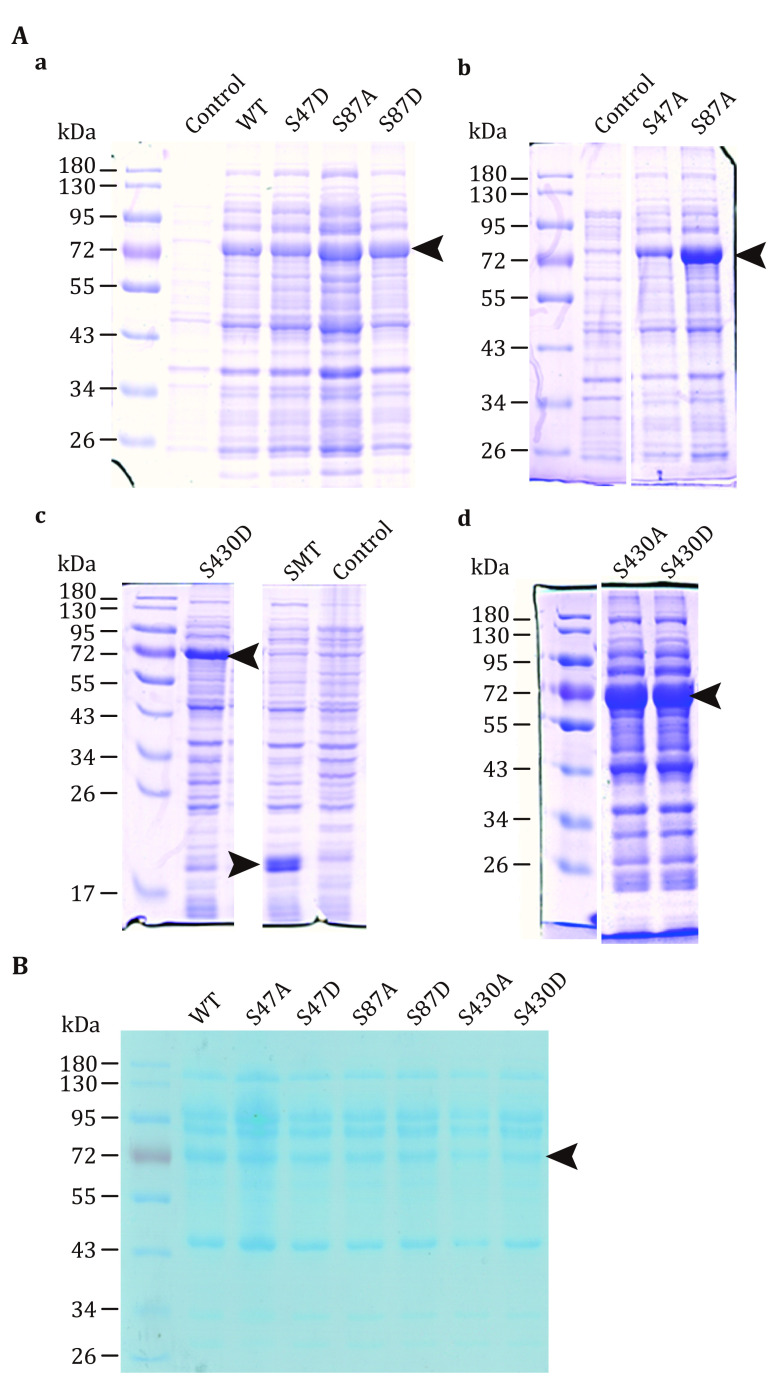
The yield of recombinant wild-type ATGL and mutants assessed by SDS-PAGE. **A** Protein expression of wild-type ATGL and mutants induced by IPTG. Wild-type ATGL, S47D, S87A and S87D (a), S47A and S87A (b), S430D and SMT3 (c), S430A and S430D (d) were expressed in bacteria cells. **B** Bacterial lysates of wild-type ATGL and mutants used for the TAG hydrolase assay. The content of ATGL and mutants was standardized by scanning the density of protein bands. See also the supplementary Fig. S1

3 Bacterial culture

(A) Expression vectors of SMT3-ATGL and ATGL mutants as well as pET-28a-SMT3-N are transformed into Transetta (DE3) competent cells.

(B) Pick five clones and culture them in 11 mL resistant LB medium at 37 °C with 200 r/min shaking until *OD*_600_ = 0.6.

(C) Transfer 1 mL of each clone to 2 mL Eppendorf tubes and add IPTG until a final concentration of 0.4 mmol/L to induce the expression of proteins at 16 °C for 24 h. Transfer 1 mL of each clone to 2 mL Eppendorf tubes and culture them under the same condition without IPTG. Preserve the rest cells at 4 °C.

(D) Collect the bacteria by centrifugation at 20,000 *g*, 4 °C for 5 min and remove the culture medium. Resuspend the bacteria with 500 μL Tris-NaCl buffer and centrifuge again to discard the supernatant.

(E) Add 200 μL 2× Sample Buffer into the bacteria and sonicate the mixture using a probe sonicator on ice for 1 min (6 s on, 6 s off) with an output power of 240 W, to prepare the samples for SDS-PAGE.

(F) Detect protein expression by SDS-PAGE and Coomassie Brilliant Blue Staining (Fig. 1A and the supplementary Fig. S1). Compare the bands of induced samples with those of uninduced samples and select clones with the strong induction.

(G) Inoculate 800 mL of resistant 2× YT medium with 8 mL of the protein expressing bacteria. Add IPTG until a final concentration of 0.4 mmol/L to induce the expression of proteins at 16 °C for 24 h.

(H) Collect the bacteria by centrifugation at 3,000 *g* for 30 min and remove the medium. Resuspended by 30 mL Tris-NaCl buffer per 800 mL bacteria medium followed by centrifugation at 3,000 *g* for 10 min and discard the supernatant.

(I) Freeze the bacteria in liquid nitrogen and stored at −80 °C.

4 Bacterial lysis

(A) Thaw the bacteria in 25 °C water bath.

(B) Sonicate the bacteria on ice in 1.6 mL Solution A per 800 mL bacteria medium for 15 min (6 s on, 6 s off) with an output power of 240 W.

(C) Centrifuge the lysate at 20,000 *g*, 4 °C for 10 min. Transfer the supernatant into PCR tubes (100 µL per tube).

(D) Take 10 µL supernatant for SDS-PAGE separation, Coomassie brilliant blue staining, and Western blot. Use ImageJ to determine the band ratio of ATGL protein which is defined using the band intensity of ATGL to divide the total band intensity of proteins in the same column. The result is used to standardize the content of ATGL in the lysate.

(E) Take 10 µL supernatant to determine the protein concentration using the BCA protein assay kit.

(F) Freeze the supernatant in liquid nitrogen and store it at −80 °C.

We find that SMT3-ATGL expression is high in Transetta (DE3) strain. Since the affinity chromatography causes an inhibited enzymatic activity of ATGL, bacterial lysates are extracted as a source of ATGL for the determination of enzymatic activity instead. The ratio of the SMT3-ATGL fusion protein in lysate should be 10%–20%.

**[CRITICAL STEP]** (4) Transform the expression vectors into Transetta (DE3) competent cells. (5) Load more uninduced sample for a better comparison. (6) Lyse the bacteria in Solution A. (7) Ensure that the concentration of ATGL in lysate is high enough. (8) Dilute the samples for Western blot.

### Adiposome preparation [TIMING 2–3 h]

This method is modified from the published protocol (Zhi* et al.* 2022).

5 Add 2.5 μmol of total phospholipid in chloroform or other solvents to a 1.5 mL Eppendorf tube, including 1,100 μg DOPC, 340 μg liver PtdIns, 520 μg DOPE (DOPC∶liver PtdIns∶DOPE = 11∶3∶5, molar ratio).

6 Mix 5 µL of TAG (roughly 4.75 mg) with 5 μL Triolein [9, 10-^3^H(N)] (0.5 μCi/μL). Dry the phospholipids and neutral lipids under a stream of N_2_.

7 Add 100 µL Buffer B and then add 5 µL (roughly 4.75 mg) of total neutral lipids in the buffer.

8 Vortex the tube for 24 cycles with 10 s on and 10 s off.

9 Centrifuge the milky lipid mixture at 20,000 *g*, 4 °C for 5 min. The fraction containing adiposomes floats at the top of the liquid. Remove the underlying solution and pellets.

10 Add 100 µL Buffer B to the fraction containing adiposomes and resuspend them by vortex. Centrifuge the sample at 1,000 *g*, 4 °C for 5 min.

11 Transfer the milky solution underneath the floating white band to a new 1.5 mL Eppendorf tube and centrifuge the sample at 1,000 *g*, 4 °C for 5 min.

12 Transfer the milky solution underneath to a new 1.5 mL Eppendorf tube and centrifuge the sample at 20,000 *g*, 4 °C for 5 min.

13 Remove the underlying solution and pellets. Resuspend the adiposomes in 100 µL Solution A. Adjust optical absorbance of adiposomes at 600 nm to *OD*_600_ = 20.

We find that the samples form a milky, homogenous solution after 24 cycles of vortex. High speed centrifugation forces the formation of pellets at the bottom of the tube. The pellet fraction contains various membranous structures. Low speed centrifugation results in the formation of white fraction on the top of emulsion. The white fraction contains large spherical structures and other amorphous structures. The purified adiposomes show as a milky solution.

**[CRITICAL STEP]** (9) The stream of N_2_ should be gentle. (10)Thorough vortex is necessary for generating the adiposomes. (11) Long pipette tip for sample loading can prevent the aggregation of adiposomes. (12) Adiposomes in Solution A should be used immediately. (13) Dispose the radioactive wastes following the instruction.

### TAG hydrolase assay [TIMING 4 h]

**Figure 2:**
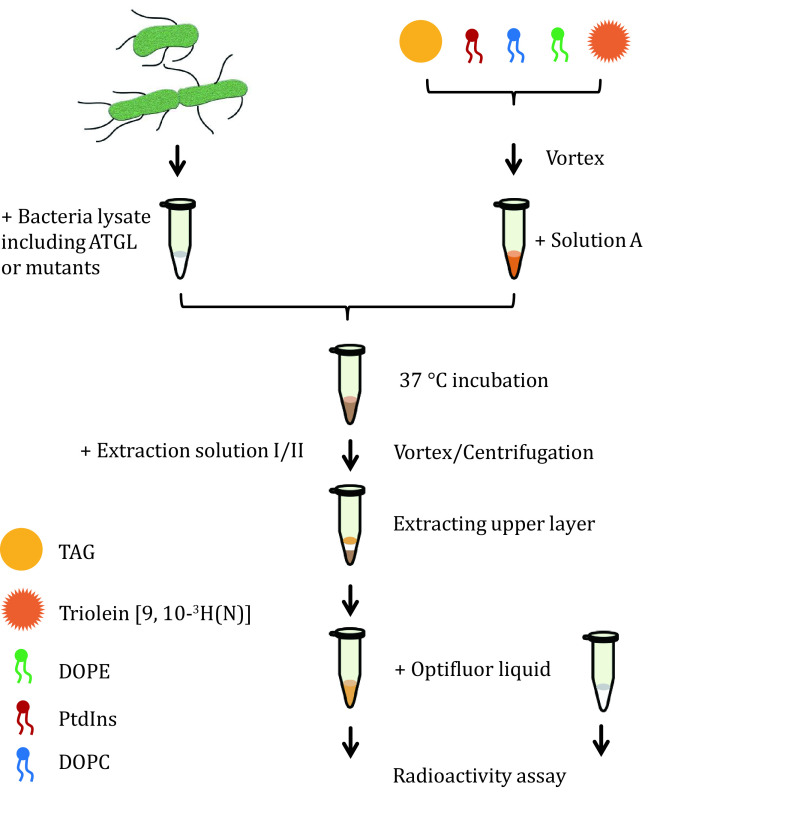
The procedure of TAG hydrolase assay. Flow diagram of measuring the enzymatic activity of ATGL using adiposome platforms. This figure is reprinted by permission from Elsevier ref. (Ma* et al.* 2021)

The overview of the procedure is provided in Fig. 2.

14 Thaw the bacterial supernatant on ice.

**Figure 3:**
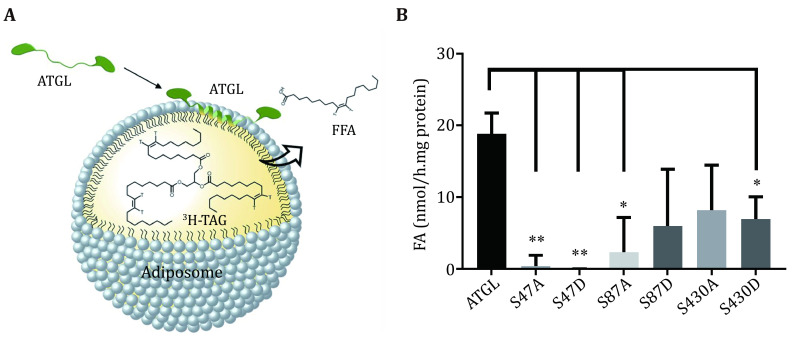
The expected outcome of the TAG hydrolase assay. **A** Scheme of ATGL binding adiposomes that are radiolabeled by triolein [9,10-^3^H(N)] and catalysing TAG hydrolysis. The radiolabeled TAG was catalyzed to release radiolabeled oleic acid. **B** The enzymatic activities of wild-type ATGL and mutants measured using adiposome platforms. Data represent mean ± s.e.m., *n* = 3. ^∗^*p* < 0.05, ^∗∗^*p* < 0.01, two-tailed *t*-test. This figure is reprinted by permission from Elsevier ref. (Ma* et al.* 2021)

15 Pipet 25 μL supernatant onto 25 μL radiolabeled adiposomes. The ATGL protein in bacterial lysates used for enzymatic activity are 1.5, 1.5, 1.0, 0.9, 0.9, 0.6, and 0.8 nmol, respectively (Fig. 1B). The mentioned doses of ATGL are set as an example. It is not necessary to follow the exact dose of ATGL for the TAG hydrolase assay. Figure 3A shows the schematic diagram of ATGL binding onto adiposomes and hydrolyzing the TAG. Use 25 μL lysate of bacteria expressing pET-28a-SMT3-N as a blank. Use 25 μL brown adipose tissue (BAT) cytosol as positive control and 25 μL Solution A as negative control. BAT cytosol is extracted following the method previously published (Yu* et al.* 2015) and dissolved in Solution A. Incubate the mixture for 1 h in a water bath at 37 °C.

16 Terminate the reaction by adding 650 µL Extraction solution I and 200 µL Extraction solution II and vortex vigorously for 5 s.

17 Centrifuge samples at 1,000 *g*, 4 °C for 10 min.

18 Transfer 200 µL of the upper aqueous phase to a scintillation vial containing 1 mL of Opti-Fluor and the radiation is measured by gamma meter.

19 Measure 25 µL radiolabeled adiposomes to determine specific substrate activity.

20 Calculate the lipase activity using the equation as following (Schweiger* et al.* 2014):

\begin{equation*}\begin{split} &
\;\;\;\;\;\;Activity\left(\mathrm{n}\mathrm{m}\mathrm{o}\mathrm{l}\;\mathrm{F}\mathrm{A}/\mathrm{m}\mathrm{g}\;\mathrm{p}\mathrm{r}\mathrm{o}\mathrm{t}\mathrm{e}\mathrm{i}\mathrm{n}\right)=\\&\;\;\;\;\;\;
\frac{\left({cpm}_{\mathrm{s}\mathrm{a}\mathrm{m}\mathrm{p}\mathrm{l}\mathrm{e}}-{cpm}_{\mathrm{b}\mathrm{l}\mathrm{a}\mathrm{n}\mathrm{k}}\right)\times \left({V}_{1}/{V}_{2}\right)}{\left({cpm}_{\mathrm{s}\mathrm{u}\mathrm{b}\mathrm{s}\mathrm{t}\mathrm{r}\mathrm{a}\mathrm{t}\mathrm{e}}/{n}_{\mathrm{F}\mathrm{A}}\right)\times {m}_{\mathrm{p}\mathrm{r}\mathrm{o}\mathrm{t}\mathrm{e}\mathrm{i}\mathrm{n}}\times 0.715t}
\end{split}\end{equation*}

where *V*_1_ is the total volume of upper water phase (2.45 mL, measured by pipette); *V*_2_ is the volume measured in the scintillation counter (0.2 mL); and *t* is the incubation time (h). *Cpm*_Sample_, *cpm*_Blank_, and *cpm*_Substrate_ are the values of counts per minute for sample, blank and substrate. *n*_FA_ is the molar value of fatty acid (nmol), *m*_Protein_ is the mass of protein (mg), *m*_Protein_ = *Ratio*_ATGL_ $ \times $ *Concentration*_Total protein_ $ \times $ 25 µL.

21 Analyze and plot the results using GraphPad prism 7.0.

The activity of mutants decreases most significantly for mutants S47A, S47D and S87A (Fig. 3B).

**[CRITICAL STEP]** (14) Assay for each sample should be repeated at least three times. (15) Keep samples on ice before transfer them in water bath. (16) Change the pipette tips for a new sample.

## ANTICIPATED RESULTS

Using this protocol, ATGL protein is expressed in bacterial cells (Fig. 1), and ATGL enzymatic activity is successfully measured on the adiposome platform (Fig. 2 and 3). There is roughly 1,900 nmol TAG in 25 μL adiposome, and the ATGL protein in bacterial lysates used for enzymatic activity are 1.5, 1.5, 1.0, 0.9, 0.9, 0.6, and 0.8 nmol, respectively (Fig. 1B). Therefore, all ATGL active sites are saturated by the substrate, and the reaction rate is determined by ATGL concentration. The enzymatic activity of ATGL and mutants is normalized through dividing the total activity by the mass of ATGL protein.

## DISCUSSION

In this study, we here provide a detailed protocol to measure ATGL activity using the adiposome platform. ATGL binds to the surface of LDs to hydrolyze the TAG, through the amphiphilic structure of the protein which will also drive the proteins to aggregate. To prevent the aggregation of ATGLs, an SMT3 tag has been added ahead of the N-terminal to increase its solubility (Wang* et al.* 2016). The S47-D166 catalytic dyad on the N-terminal of ATGL is critical for TAG hydrolysis (Duncan* et al.* 2010), while both S47A and S47D decrease the enzymatic activity *in vitro* and *in vivo* (Ma* et al.* 2021). Through site-directed mutation, phosphorylation site mutants of ATGL have been constructed. However, the purified ATGL shows a low lipase activity. BAT cytosol is used to study the factors affecting ATGL enzymatic activity *in vitro*, since it is rich in highly active ATGLs (Yu* et al.* 2015). High concentrations of imidazole, absence of PtdIns and Buffer B result in a decreased enzymatic activity of ATGL in BAT cytosol (data not shown). Subsequently, bacterial lysate expressing ATGL has been used as the source of ATGL to characterize its activity.

Radiolabeled or fluorogenic substrates have been used to measure ATGL activity. The efficiencies of existing methods are compared in Table 9. Lipid emulsion and micelle are prepared with triolein and ^3^H-triolein by sonication, the radiation of which is 50 times and 150,000 times higher than that of adiposome respectively. According to the method modified from the published paper (Ma* et al.* 2021; Schweiger* et al.* 2014), the radiation of lipid emulsion is roughly 35.71 µCi/mg triolein, whereas that of adiposomes is 0.53 µCi/mg TAG. Purified LDs are isolated from cells labeled using ^3^H-oleate. Despite the drawback of time-consuming, the radiation of TAG in LDs is more rational than lipid emulsions and micelles but is still twice higher than that of adiposome. Since the exact amounts of ATGL proteins in the cell lysates are unknown, it is difficult to compare the sensitivity of different methods. However, extreme overexpression causes the death of mammalian cells, hence it is supposed that ATGL expression is higher in bacterial cells, which might be one of the reasons that less radiation is required in our method. To ensure the homogeneity of adiposome, 24 times of vortex and two rounds of purification are conducted, resulting in a longer time than emulsion and micelle in substrate preparation. As a result, adiposome resembles the natural LDs recruiting ATGL to the phospholipid surface, inducing ATGL to form correct conformation. Considering substrate availability, it is also more accurate to measure ATGL activity using adiposome with an intact phospholipid monolayer over the neutral lipid core.

**Table 9:** Comparison of ATGL activity assays

Substrate	Label	Radiation	Time	Reference
Lipid emulsion	^3^H-triolein	40,000 cpm/nmol triolein	3 h	(Schweiger* et al.* 2008; Zimmermann* et al.* 2004)
Micelle	^3^H-triolein	117,660,000 cpm/nmol triolein	3 h	(Duncan* et al.* 2008)
Purified LDs	^3^H-oleate	1,660 cpm/nmol TAG	3 d	(Schweiger* et al.* 2008)
Adiposome	^3^H-triolein	752 cpm/nmol TAG	4 h	(Ma* et al.* 2021)
NBD-TAG vesicle	NBD-TAG	8,000 fluorescence units/nmol TAG	8 h	(Rajan* et al.* 2021)

This method does present some limitations. First, the use of radioactive substrate limits the suitability of this assay for high-throughput screening. Latest publication reports a new fluorescence method to measure the lipase activity without separating substrates from products (Rajan* et al.* 2021). As shown in Table 9, although it costs twice longer time to use NBD-TAG than ^3^H-triolein owing to the preparation of a standard curve, separation of products from substrates as well as reaction termination are not required and the fluorescent signal can be scanned by a microplate reader. These advantages make the fluorescence-based method suitable for measuring real-time enzyme kinetics and high-throughput screening. It is probably feasible to replace radiolabeled triolein with NBD-labeled TAG to construct adiposome, and detect the fluorescent signal in a 96- or 384-microwell plate in the following research. Second, the absence of BSA causes the loss of generated FFAs from TAG hydrolysis. BSA is widely used as a carrier to bind the generated FFAs, while BSA addition causes a reduced ATGL activity using the adiposome platform. It is speculated that BSA might hinder ATGL to target adiposomes. Third, the amount of ATGL protein in bacterial lysates is estimated by grey scale scanning of stained gels. Recently, the purified ATGL variant covering residues M1 to D288 demonstrates an improved lipolytic activity (Kulminskaya* et al.* 2021), which can be applied in the adiposome platform for accurate detection of ATGL activity.

Through manipulating the lipids or proteins of the adiposome platform, this method could be applied in other research. For example, this method could be applied to measure the activity of other TAG hydrolases, such as hormone-sensitive lipase (HSL). Despite phosphorylation, protein–protein interaction may also regulate ATGL enzymatic activity. This method is suitable to investigate modulators that affect ATGL activity. By using radiolabeled phospholipids or cholesterol ester to prepare adiposome, this method could be adapted to measure the activity of phospholipases and cholesterol ester hydrolases.

In conclusion, this protocol validates the adiposome as a suitable platform for the enzymatic activity study of ATGL* in vitro*.

## Conflict of interest

Xuejing Ma, Zelun Zhi, Shuyan Zhang and Pingsheng Liu declare that they have no conflict of interest.
